# Conserved Orb6 Phosphorylation Sites Are Essential for Polarized Cell Growth in *Schizosaccharomyces pombe*

**DOI:** 10.1371/journal.pone.0037221

**Published:** 2012-05-21

**Authors:** Guohong Liu, Dallan Young

**Affiliations:** Departments of Biochemistry & Molecular Biology and Oncology, University of Calgary, Calgary, Alberta, Canada; Institute of Developmental Biology and Cancer Research, France

## Abstract

The Ndr-related Orb6 kinase is a key regulator of polarized cell growth in fission yeast, however the mechanism of Orb6 activation is unclear. Activation of other Ndr kinases involves both autophosphorylation and phosphorylation by an upstream kinase. Previous reports suggest that the Nak1 kinase functions upstream from Orb6. Supporting this model, we show that HA-Orb6 overexpression partially restored cell polarity in *nak1* ts cells. We also demonstrated by coimmunoprecipitation and *in vitro* binding assays that Nak1 and Orb6 physically interact, and that the Nak1 C-terminal region is required forNak1/Orb6 complex formation *in vivo*. However, results from *in vitro* kinase assays did not show phosphorylation of recombinant Orb6 by HA-Nak1, suggesting that Orb6 activation may not involve direct phosphorylation by Nak1. To investigate the role of Orb6 phosphorylation and activity, we substituted Ala at the ATP-binding and conserved phosphorylation sites. Overexpression of kinase-dead HA-Orb6^K122A^ in wild-type cells resulted in a loss of cell polarity, suggesting that it has a dominant-negative effect, and it failed to rescue the polarity defect of *nak1* or *orb6* ts mutants. Recombinant GST-Orb6^S291A^ did not autophosphorylate *in vitro* suggesting that Ser291 is the primary autophosphorylation site. HA-Orb6^S291A^ overexpression only partially rescued the *orb6* polarity defect and failed to rescue the *nak1* defect, suggesting that autophosphorylation is important for Orb6 function. GST-Orb6^T456A^ autophosphorylated *in vitro*, indicating that the conserved phosphorylation site at Thr456 is not essential for kinase activity. However, HA-Orb6^T456A^ overexpression had similar effects as overexpressing kinase-dead HA-Orb6^K122A^, suggesting that Thr456 is essential for Orb6 function *in vivo*. Also, we found that both phosphorylation site mutations impaired the ability of Myc-Nak1 to coimmunoprecipitate with HA-Orb6. Together, our results suggest a model whereby autophosphorylation of Ser291 and phosphorylation of Thr456 by an upstream kinase promote Nak1/Orb6 complex formation and Orb6 activation.

## Introduction

Cell polarity underlies many cellular processes important for morphogenesis, differentiation, growth and motility in eukaryotic cells [1]. Fission yeast grow at their cell ends in a cell cycle regulated program to form an elongated cell shape [2], and thus provide an excellent model for studying the mechanisms that determine cell polarity [3], [4]. A previous study identified 12 temperature sensitive (ts) “orb” mutants that are defective in polarized cell growth and exhibit a spherical cell morphology [5]. Our lab identified Nak1 and showed that it is essential for cell growth and polarity in fission yeast [6], and *nak1* was subsequently shown to be allelic with *orb3* [7]. Nak1 belongs to the group III germinal center kinase (GCK) family, which comprises protein Ser/Thr kinases related to human GCK and belongs to the Ste20-like kinase superfamily [8]. More recent studies suggest that Nak1, Orb6 and several other proteins form the MOR signaling network that regulates cell polarity and cell separation [9], and is related to the RAM network in budding yeast [10]. The RAM network comprises six proteins (Hym1, Kic1, Cbk1, Tao3, Mob2, and Sog2) and homologs of several of these components have been identified in fission yeast (Pmo25, Nak1, Orb6, Mor2, and Mob2) and other organisms, suggesting that a RAM-like network is conserved among eukaryotic organisms.

Studies suggest that Orb6 is the critical downstream component of the MOR pathway that regulates cell polarity by spatially regulating the localization of Cdc42 [11], [12]. Orb6 is a Ser/Thr kinase belonging to the conserved Ndr (nuclear Dbf2-related) kinase family [11], [13]. The Ndr family of protein kinases share a conserved N-terminal domain that interacts with Mob proteins [14], and have conserved sites for autophosphorylation and phosphorylation by an upstream kinase [13], [15]. In several cases, genetic and biochemical evidence indicate that Ste20-like kinases are the upstream kinases that phosphorylate Ndr kinases in budding yeast [16], Drosophila [17], and mammalian cells [18]–[20]. Previous reports suggest that activation of Ndr kinases is a multistep process involving binding of Mob proteins and phosphorylation at the conserved sites [13], [14], and the conserved phosphorylation sites have been shown to be important for the function of several Ndr kinases [15], [16], [21]–[23]. The mechanism of Orb6 activation remains unclear, although it was reported that Orb6 interacts with Mob2 [24], and Orb6 kinase activity is dependent on the MOR network components Pmo25, Nak1 and Mor2 [9]. In this paper, we have further investigated the relationship of Nak1 and Orb6, and the role of conserved phosphorylation sites in Orb6 function.

## Results

### Overexpression of Orb6 partially rescues the *nak1* ts polarity defect

**Figure 1:**
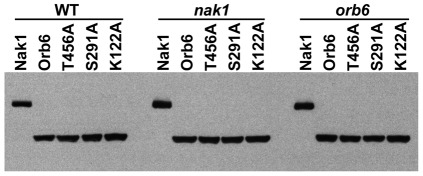
Expression of Nak1 and Orb6 mutants. HA-Nak1, HA-Orb6, HA-Orb6^T456A^, HA-Orb6^S291A^, and HA-Orb6^K122A^ were expressed in WT (SP199), *nak1* and *orb6* ts cells using plasmids derived from pREP3X containing the thiamine repressible *nmt1* promoter (Materials and Methods). HA-tagged proteins were detected by Western blots using anti-HA (12CA5) monoclonal antibody.

**Figure 2:**
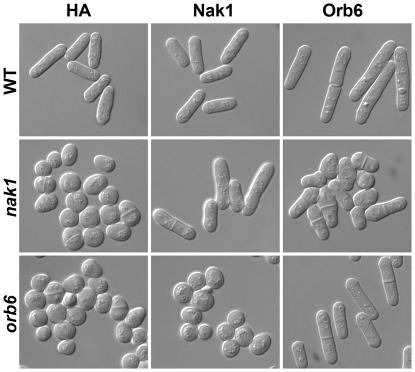
Orb6 overexpression partially rescues the *nak1* ts polarity defect. HA-Nak1 or HA-Orb6 were expressed in WT (SP199), *nak1* and *orb6* ts cells as described in Figure 1. Cells were grown in thiamine free liquid PMAU media to an O.D.  = 1.0 at 33°C and examined by DIC microscopy.

To investigate the functional relationship of Nak1 and Orb6 in the MOR pathway, we performed overexpression studies. As mentioned above, *orb* ts mutants exhibit a spherical cell shape, indicating a loss of cell polarity [5]. We looked for the ability of overexpressed proteins to rescue this defect. We overexpressed HA-Nak1 and HA-Orb6 in wild type, *nak1* ts, and *orb6* ts strains. The levels of HA-proteins expressed in these strains were similar as detected by western blots (Figure 1). We found that HA-Nak1 overexpression restored normal cell shape in *nak1* ts cells, but not in *orb6* ts cells at 33°C (Figure 2). As previously reported, Orb6 overexpression in WT cells resulted in an increased cell length at division, indicating a delay in the onset of mitosis [11]. Overexpression of HA-Orb6 restored polarity in *orb6* cells, and also partially restored polarity in *nak1* cells. These observations are consistent with a model where Orb6 functions downstream from Nak1 to regulate cell polarity.

### Nak1 and Orb6 can physically interact

**Figure 3:**
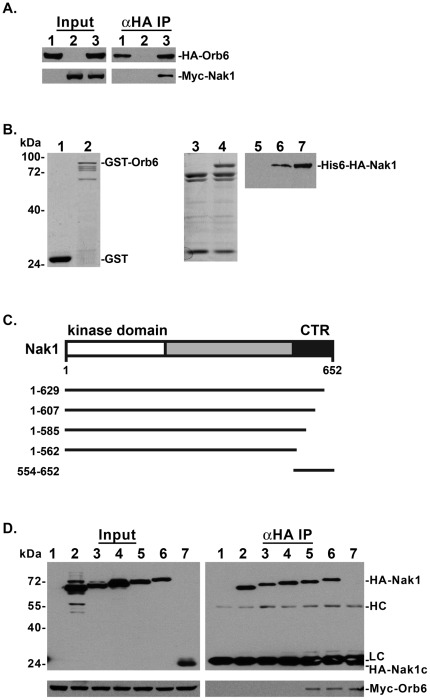
Nak1 and Orb6 interact *in vitro* and *in vivo*. A. Extracts from WT (SP199) cells expressing HA-Orb6 and control vector (lane 1), Myc-Nak1 and control vector (lane 2), or HA-Orb6 and Myc-Nak1 (lane 3) from *nmt1* promoter expression plasmids were analyzed by western-blots using anti-Myc (9E10) or anti-HA (12CA5) monoclonal antibodies (Input panels). HA-Orb6 was immunoprecipitated from cell extracts with anti-HA antibody and equal portions of the immunoprecipitates were probed with anti-HA antibody and anti-Myc antibody (αHA IP panels). **B.** Interaction of purified recombinant GST-Orb6 and His_6_-HA-Nak1 was assayed by an *in vitro* binding assay (Materials and Methods). The left panel shows Coomassie blue staining of purified recombinant GST (lane 1), GST-Orb6 (lane 2), His_6_-vector control (lane 3), His_6_-HA-Nak1 (lane 4). Purified His_6_-HA-Nak1 (input, lane 7) was incubated with either GST (lane 5) or GST-Orb6 (lane 6) bound to Glutathione Sepharose 4B beads. The right panel shows a Western blot using anti-HA (12CA5) antibody of His_6_-HA-Nak1 bound to the beads. **C.** Schematic diagram of mutant Nak1 expression constructs. The Nak1 N-terminal kinase domain (residues 1–262) and C-terminal (CTR) region (554–652) are indicated. The numbers at the left and the bars at the right indicate the region of Nak1 encoded by the various deletion constructs. **D.** Extracts from wild-type (SP199) cells co-expressing Myc-Orb6 with the control vector (lane 1), HA-Nak1^1–562^ (lane 2), HA-Nak1^1–585^ (lane 3), HA-Nak1^1–607^ (lane 4), HA-Nak1^1–629^ (lane 5), HA-Nak1 (lane6), or HA-Nak1^554–652^ (lane 7) were analyzed by western-blots using anti-Myc (9E10) or anti-HA (12CA5) monoclonal antibodies (Input panels). Extracts were immunoprecipitated with anti-HA antibody and equal portions of the immunoprecipitates were probed with anti-HA antibody and anti-Myc antibody (αHA IP panels).

It was previously reported that Orb6 and Nak1 interact in a yeast two-hybrid test [9]. To verify that these proteins can physically interact, we performed coimmunoprecipitation experiments using extracts of WT cells expressing HA-Orb6 and Myc-Nak1 (Figure 3A). Our results show that Myc-Nak1 co-immunoprecipitated with HA-Orb6 (lane 3), but not with the control (lane 2). To further investigate the interaction between Nak1 and Orb6, we performed *in vitro* binding assays using recombinant proteins. First, we expressed and purified recombinant His_6_-HA-Nak1 and GST-Orb6 from *E. coli*, and then performed GST pull-down assays (Figure 3B). We found that His_6_-HA-Nak1 bound to purified GST-Orb6 (lane 6) but not to GST (lane 5), indicating that Nak1 and Orb6 interact directly *in vitro*.

To determine which regions of Nak1 are required for the interaction with Orb6, we generated expression constructs containing a series of deletions within the Nak1 coding sequence (Figure 3C). Then we co-expressed these HA-Nak1 deletion mutants with Myc-Orb6 in WT cells. We found that Myc-Orb6 co-immunoprecipitated with HA-Nak1, HA-Nak1^1–629^ and HA-Nak1^554–652^, but not with HA-Nak1^1–562^, HA-Nak1^1–585^ or HA-Nak1^1–607^ (Figure 3D). These results indicate that a region (aa 608–629) near the Nak1 C-terminus is required for Nak1/Orb6 complex formation *in vivo*.

### Nak1 does not phosphorylate purified recombinant Orb6 *in vitro*

**Figure 4:**
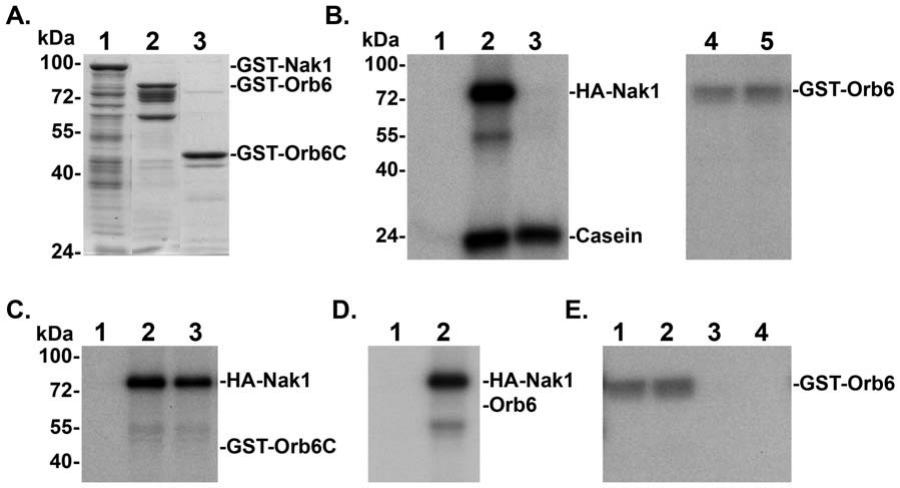
Orb6 phosphorylation *in vitro*. **A.** Coomassie blue staining of purified recombinant GST-Nak1 (lane 1), GST-Orb6 (lane 2), GST-Orb6^301–469^ (GST-Orb6C) (lane 3). **B.** Kinase assays were performed using purified recombinant GST-Orb6 and immunoprecipitated HA-Nak1 from yeast cell extracts (Materials and Methods). Protein A-Sepharose beads bound with HA-Nak1 were removed from samples in lanes 3, 5 following the kinase reaction. HA + Casein (lane 1), HA-Nak1 + Casein (lane 2), HA-Nak1 + Casein (lane 3), HA + GST-Orb6 (lane 4), HA-Nak1 + GST-Orb6 (lane 5). **C.** Kinase assays were performed using purified recombinant GST-Orb6C and immunoprecipitated HA-Nak1 from yeast cell extracts. HA (lane 1), HA-Nak1 (lane 2), HA-Nak1 + GST-Orb6C (lane 3). **D.** Kinase assays were performed using purified Orb6 lacking GST and immunoprecipitated HA-Nak1. HA + Orb6 (lane 1), HA-Nak1 + Orb6 (lane 2). **E.** Kinase assays were performed using purified recombinant GST-Orb6 (lane 1), GST-Orb6^T456A^ (lane 2), GST-Orb6^S291A^ (lane 3) and GST-Orb6^K122A^ (lane 4).

Our results along with previously reported evidence that Orb6 kinase activity is severely reduced in *nak1* ts cells [9] suggest that Orb6 functions downstream from Nak1. To investigate if Orb6 is a substrate of Nak1, we performed *in vitro* kinase assays. First, GST-Nak1, GST-Orb6 and the C-terminal region GST-Orb6^301–469^ (GST-Orb6C) were expressed in *E. coli.* and purified (Figure 3A). We then performed *in vitro* kinase assays to check if recombinant GST-Nak1 and GST-Orb6 were active. It was previously reported that GST-Orb6 from fission yeast extract can autophosphorylate [25]. We similarly found that purified recombinant GST-Orb6 autophosphorylated indicating that it was active (Figure 4B, lane 4). In contrast, recombinant GST-Nak1 failed to autophosphorylate or phosphorylate casein (not shown), indicating that it was not active. Since recombinant GST-Nak1 was not active, we used immunoprecipitates of HA-Nak1 from yeast cell extracts for kinase assays. As we previously reported [6], HA-Nak1 autophosphorylates and can phosphorylate casein *in vitro* (Figure 4B, lane 2). However, since HA-Nak1 and GST-Orb6 migrate at similar positions, autophosphorylated HA-Nak1 would mask any phosphorylation of GST-Orb6. Therefore, HA-Nak1 was removed following the kinase reactions (Figure 4B, lanes 3, 5). We found that the presence of HA-Nak1 in the kinase reactions did not significantly increase the level of GST-Orb6 phosphorylation above the level due to GST-Orb6 autophosphorylation (lanes 4, 5). We also tested if HA-Nak1 would phosphorylate the Orb6 C-terminal fragment (GST-Orb6C) containing the conserved site for phosphorylation by an upstream kinase at Thr456. Since GST-Orb6C is much smaller than HA-Nak1, we did not need to remove HA-Nak1 following the kinase reaction. We did not detect phosphorylatation of GST-Orb6C by HA-Nak1 (Figure 4C).

In order to separate the signals from phosphorylated HA-Nak1 and Orb6, we also cleaved the GST moiety from GST-Orb6 prior to performing the kinase assay. Interestingly, we found that purified Orb6 lacking GST no longer autophosphorylated (Figure 4D, lane 1), suggesting that GST-mediated dimerization may be necessary for efficient Orb6 autophosphorylation. Since the mobility of Orb6 and HA-Nak1 could be distinguished, we did not remove HA-Nak1 following the kinase reactions. Again, we did not observe any phosphorylation of Orb6 due to the presence of HA-Nak1 in the kinase reaction (Figure 4D). Together our evidence does not support a conclusion that HA-Nak1 can phosphorylate GST-Orb6 *in vitro*. Although, it is possible that other factors (e.g. Mob2 and Mor2) are required to facilitate Nak1 phosphorylation of Orb6.

### The conserved phosphorylation sites at Ser291 and Thr456 are required for Orb6 function

Ndr kinases share conserved sites for both autophosphorylation and phosphorylation by a regulatory upstream kinase. By sequence alignment with other Ndr kinases, Orb6 was found to have corresponding conserved sites at Ser291 and Thr456 respectively [13], [15]. To investigate the potential phosphorylation and role of these sites, we introduced Ala substitutions at these sites, and expressed and purified recombinant GST-Orb6^S291A^ and GST-Orb6^T456A^. We also constructed and purified a kinase-dead GST-Orb6^K122A^ containing an Ala substitution at the invariant catalytic lysine residue in the ATP binding site that is critical for activity of all protein kinases [26]. We then performed *in vitro* kinase assays to determine whether these purified recombinant mutant proteins were active. We found that only GST-Orb6 and GST-Orb6^T456A^ were autophosphorylated, while phosphorylation of GST-Orb6^K122A^ and GST-Orb6^S291A^ was not detected (Figure 4E). These observations indicate that Orb6^K122A^ lacks kinase activity, and suggest that Ser291 is the primary autophosphorylation site, while Thr456 phosphorylation is not required for kinase activity. Although, it is also possible that the S291A mutation inhibits the kinase activity.

**Figure 5:**
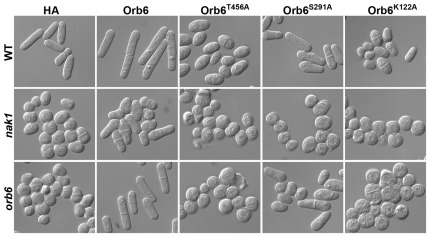
Conserved phosphorylation sites are essential for Orb6 function. HA-Orb6, HA-Orb6^T456A^, HA-Orb6^S291A^ or HA-Orb6^K122A^ were expressed in WT (SP199), *nak1* and *orb6* ts cells using plasmids derived from pREP3X containing the thiamine repressible *nmt1* promoter. Cells were grown in thiamine free liquid PMAU media to an O.D.  = 1.0 at 33°C and examined by DIC microscopy.

To examine the function of these conserved sites, we expressed HA-Orb6^K122A^, HA-Orb6^S291A^, and HA-Orb6^T456A^ in WT, *nak1* and *orb6* ts strains. We found that all of these HA-Orb6 mutants were stably expressed at similar levels in these cells (Figure 1). Then we examined the ability of overexpression of these proteins to rescue the polarity defects of *nak1* and *orb6* ts strains (Figure 5). We found that overexpression of the kinase-dead HA-Orb6^K122A^ made WT cells spherical, suggesting that this mutation results in a dominant-negative effect. Also, HA-Orb6^K122A^ overexpression could not rescue the polarity defect of either *nak1* or *orb6* strains suggesting that Orb6 kinase activity is essential for its normal function. We also found that HA-Orb6^T456A^ overexpression made WT cells spherical or pear shaped, which is very similar to the effect of overexpressing HA- Orb6^K122A^, suggesting that Orb6^T456A^ is a non-functional mutant and has a dominant negative effect. Consistent with this, overexpression of HA-Orb6^T456A^ could not rescue *nak1* and *orb6* polarity defects. In contrast, overexpression of HA-Orb6^S291A^ did not significantly affect the WT cell shape, but only partially rescued the *orb6* polarity defect and failed to rescue the *nak1* polarity defect. These results suggest that autophosphorylation of Ser291 is important for normal Orb6 function, while phosphorylation of Thr456 by an upstream kinase is essential for Orb6 function.

**Figure 6:**
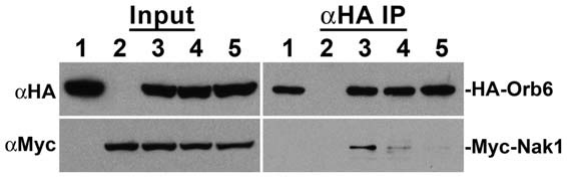
Orb6 phosphorylation site mutants impair interaction with Nak1. Extracts from WT (SP199) cells expressing HA-Orb6 and control vector (lane 1), Myc-Nak1 and control vector (lane 2), HA-Orb6 and Myc-Nak1 (lane 3), HA-Orb6 ^S291A^ and Myc-Nak1 (lane 4), or HA-Orb6^T456A^ and Myc-Nak1 (lane 5) from *nmt1* promoter expression plasmids were analyzed by western-blots using anti-Myc (9E10) or anti-HA (12CA5) monoclonal antibodies (Input panels). HA-Orb6 was immunoprecipitated from cell extracts with anti-HA antibody and equal portions of the immunoprecipitates were probed with anti-HA antibody and anti-Myc antibody (αHA IP panels).

Since the conserved phosphorylation site mutants failed to function normally *in vivo*, we examined whether phosphorylation of these sites are important for the interaction of Orb6 with Nak1. To test this idea, we performed co-immunoprecipitation experiments using cells co-expressing Myc-Nak1 and either HA-Orb6, HA-Orb6^S291A^ or HA-Orb6^T456A^. Interestingly, we found that the amounts of Myc-Nak1 that co-immunoprecipitated with HA-Orb6^S291A^ or HA-Orb6^T456A^ were significantly less than with HA-Orb6 (Figure 6). Therefore, Ser291 and Thr456 are important for the interaction of Orb6 with Nak1 *in vivo*, and phosphorylation of these sites may promote this interaction.

## Discussion

While studies have shown that phosphorylation is involved in activating Ndr kinases in several organisms, the roles of specific phosphorylation sites in Orb6 have not been reported. As mentioned above, sites for autophosphorylation and phosphorylation by upstream kinases are conserved among Ndr kinases including Orb6. We explored the roles of these phosphorylation sites and kinase activity in Orb6 function. We found that substitution of the catalytic lysine residue with Ala abolished Orb6 kinase activity, as previously shown for human Ndr [27]. Since kinase-dead Orb6^K122A^ failed to rescue the *nak1* or *orb6* polarity defects, Orb6 kinase activity appears to be critical for the ability of Orb6 to promote cell polarity. This is consistent with a previous report that inhibition of Orb6 kinase activity alters Cdc42 localization at the cell tips, which is required to promote polarized cell growth [12]. Also, the polarity defect due to HA-Orb6^K122A^ overexpression in WT cells suggests that this mutant protein may dominantly interfere with the MOR pathway by binding to either an upstream regulator or downstream target.

The inability of GST-Orb6^S291A^ to autophosphorylate strongly suggests that the conserved Ndr autophosphorylation site at Ser291 is the primary autophosphorylation site in Orb6. Although, it is possible that this mutation renders the Orb6 kinase inactive. While this site is conserved with known autophosphorylation sites in other Ndr kinases, we do not have direct evidence that Ser291 is a site for autophosphorylation *in vivo*. It is also possible that this site is phosphorylated by another kinase. Since expression of this mutant kinase only partially rescued the *orb6* polarity defect, it appears that autophosphorylation is important for Orb6 function. While the conserved site at Thr456 does not appear to affect kinase activity *in vitro*, it is essential for Orb6 function *in vivo*, suggesting that phosphorylation of this site by an upstream kinase is a key step in the regulation of Orb6 activity. Similarly, the conserved phosphorylation site in the budding yeast Ndr-related Cbk1 kinase is required for Cbk1 function, but not for kinase activity [28]. Our observations are consistent with previous reports that Ndr activation involves both autophosphorylation and phosphorylation by an upstream kinase [13], [15].

Previous studies suggest that Nak1 functions upstream of Orb6 in the MOR pathway. First, both proteins are essential for cell growth and polarity, suggesting that they may function in the same pathway. Also, it was reported that Orb6 kinase activity is severely reduced in a *nak1* ts strain, whereas Nak1 activity is not impaired in an *orb6* mutant, further suggesting that Nak1 functions upstream from Orb6 [9]. Our observation that Orb6 overexpression partially rescued the *nak1* ts polarity defect is consistent with this model. Together, these observations along with reports that Ste20-like kinases phosphorylate other Ndr kinases suggest the possibility that Nak1 regulates Orb6 by phosphorylation of Thr456. However, our results from *in vitro* kinase assays suggest that the activation of Orb6 may not involve direct phosphorylation by Nak1, although it is possible that other factors such as Mob2 and Mor2 may be required to facilitate Orb6 phosphorylation. If Nak1 does not phosphorylate Thr456, then it is possible that another Ste20-like kinase regulates Orb6 by phosphorylating this site. Since overexpression of Orb6 partially suppressed the *pak1/orb2* ts phenotype, it has been suggested that Pak1 kinase functions upstream from Orb6 [11].

If Nak1 does not phosphorylate Orb6, then perhaps it affects Orb6 activity by another mechanism, either indirectly by modulating another Orb6 regulator, or directly through its interaction with Orb6. Our evidence indicates that Orb6 and Nak1 can physically interact, and that the Nak1 C-terminal region is required for this interaction. While it was previously reported that Nak1 and Orb6 interact in a yeast two-hybrid test, the same study reported that Nak1-GFP and Orb6-HA failed to coimmunoprecipitate [9]. Since the Nak1 C-terminal region is required for this interaction, it is possible that the C-terminal tags interfered with the ability of the proteins to interact in that study. Since Orb6 and Nak1 both localize to sites of cell growth and division [7], [11] and can physically interact, they may function together in a complex at these sites. Both Nak1 and Orb6 have been reported to interact with Mor2 [9], and a Nak1-Orb6 fusion can rescue a *mor2* mutation suggesting Mor2 may act as a scaffold to bring Orb6 and Nak1 together [29]. The Nak1-Orb6 fusion can also bypass the inhibition of MOR by SIN suggesting that interaction between Nak1 and Orb6 is a key regulatory step in the activation of Orb6. However, our result that Nak1 and Orb6 can directly interact indicates that Mor2 is not necessary for this interaction *in vitro*, but it may be important to facilitate this interaction *in vivo*. Since the Nak1 C-terminal region is required for the interaction of Nak1 with other proteins in addition to Orb6 [30], perhaps Mor2 is required to specifically promote the formation of a complex with Orb6. Since Ala substitutions of the conserved phosphorylation sites impair both Orb6 function and its interaction with Nak1, phosphorylation of these sites may be key mechanisms that regulate this interaction and Orb6 activation. Thus, we propose a model whereby Orb6 autophosphorylation at Ser291 and phosphorylation of Thr456 by a regulatory kinase promote Nak1/Orb6 complex formation and Orb6 activation.

## Materials and Methods

### Yeast Strains and methods

The *S. pombe* strain SP199 (*h+ leu1–32 ura4–d18 ade6-M210*) was obtained from Dr. David Beach; the *orb3* (*h+ orb3–167 leu1–32 ade-M216*) and *orb6* (*h- orb6-25 leu1–32 ade6-M210*) ts strains were obtained from Dr. Paul Nurse [5]. Methods for yeast culture, transformation and genetic analyses were described previously [31], [32].

### Plasmids

Procedures for DNA cloning and analysis have been previously described [33]. pRep3X-HA, pRep3XC-HA, pRep4X-Myc, and pRep4X-Myc-Nak1 have been previously described [6], [30], [34]. pRep3X-HAOrb6 was constructed by inserting a PCR-amplified genomic DNA fragment encoding Orb6 into the SpeI/XmaI sites of pRep3X-HA. pRep3XC-HANak1, pRep3XC-HANak1^1–562^, pRep3XC-HANak1^1–585^, pRep3XC-HANak1^1–607^, and pRep3XC-HANak1^1–629^ were constructed by inserting a PCR-amplified genomic DNA fragments encoding the corresponding regions of Nak1 into the SpeI/NotI sites of pRep3XC-HA. pRep3X-HANak1^554–652^ was constructed by inserting a PCR-amplified genomic DNA fragment encoding the corresponding region of Nak1 into the SpeI/BamHI site of pRep3X-HA. pET33b-His6-HA-Nak1 was constructed by inserting HA-Nak1 into the BamHI/SalI site of pET33b. pGEX-5X-1-Nak1 was constructed by inserting a PCR-amplified cDNA fragment encoding Nak1 into the BglII/NotI sites of pGEX-5X-1. pGEX-5X-1-Orb6 and pGEX-5X-1-Orb6 ^301–469^ were constructed by inserting PCR-amplified cDNA fragments encoding the corresponding regions of Orb6 into the BglII/NotI sites of pGEX-5X-1. pGEX-6p-1-Orb6 was constructed by inserting a PCR-amplified cDNA fragment encoding Orb6 into the BamHI/NotI site of pGEX-6p-1. Point mutations in pGEX-5X-1-Orb6 were made using the QuickChange Lightning Site-Directed Mutagenesis Kit (Stratagene) to produce pGEX-5X-1-Orb6^K122A^, pGEX-5X-1-Orb6^S291A^, and pGEX-5X-1-Orb6^T456A^. Similarly, point mutations in pRep3X-HAOrb6 were made to produce pRep3X-HAOrb6^K122A^, pRep3X-HAOrb6^S291A^, and pRep3X-HAOrb6^T456A^.

### Recombinant protein purification

His_6_-HA-Nak1 was expressed in *E. coli* BL21 cells and purified by binding to NiNTA agarose beads (Qiagen) and eluting with NiNTA elution buffer (50 mM NaH_2_PO_4_, 300 mM NaCl, 250 mM imidazole, 0.2 mM PMSF, 1 µg/ml pepstatin, 1 µg/ml leupeptin). The eluted protein was concentrated and imidazole was removed using a Vivaspin 6 ultrafiltration device (30,000 MWCO PES, GE Healthcare).

GST fusion proteins were expressed in *E. coli* BL21 from pGEX-5X-1 or pGEX-6p-1 and purified by binding to Glutathione Sepharose 4B beads (GE Healthcare Life Sciences) and eluting with GST elution buffer (50 mM Tris pH8.0, 20 mM Glutathione, 1 mM DTT, 0.2 mM PMSF, 0.2 µg/ml pepstatin, 0.2 µg/ml leupeptin). The eluted protein was concentrated using a Vivaspin 6 ultrafiltration device (30,000 MWCO PES, GE Healthcare). GST was removed from purified recombinant GST-Orb6 following cleavage with PreScission Protease (GE Healthcare) at 4°C for 12 h.

### *In vitro* binding assay

GST-Orb6 bound to Glutathione Sepharose 4B beads were incubated with BL21 cell lysate (2 mg protein) containing His_6_-HANak1 for 2 h. The beads were washed 3 times with wash buffer (150 mM NaCl, 1 mM DTT, 0.2 mM PMSF, 16 mM Na_2_HPO_4_, 4 mM NaH_2_PO_4_, pH 7.3), and were divided evenly into two parts. The samples were resuspended in SDS-PAGE sample buffer and boiled for 5 min, separated on SDS-PAGE gels, and then stained with Coomassie Blue or analyzed by western blots using anti-HA (12CA5) antibody.

### Immunoprecipitation and kinase assays

Immunoprecipitation and *in vitro* kinase assays were performed as previously described [6]. Yeast were grown in minimal media to an OD_600_  = 0.8. Cells were collected by centrifugation and resuspended in lysis buffer (50 mM Tris-HCl pH 7.4, 100 mM NaCl, 2 mM EDTA, 0.1% Non-Idet P-40, 10 µg/ml leupeptin, 1 µg/ml pepstatin A, 100 µg/ml PMSF, 1 µg/ml aprotinin). Cells were vortexed with glass beads, and crude lysates were cleared by centrifugation at 14,000 rpm in a microfuge for 15 min. Relative protein concentrations were determined by Bio-Rad protein assay. Cell lysates were pre-cleared with protein A sepharose slurry for 20 min, and then incubated with 12CA5 (anti-HA) antibody and protein A sepharose for 2 hours at 4°C. Immunoprecipitates were centrifuged at 1,000 rpm in a microfuge, and washed three times with 750 μl yeast lysis buffer; samples were divided evenly upon the last wash. Half of the samples were analyzed by western blots with 12CA5 antibody to detect HA-epitope tagged proteins. The other half of the immunoprecipitates were resuspended in kinase buffer (50 mM Tris-HCl pH 7.4, 100 mM NaCl, 10 mM MgCl_2_, 1 mM MnCl_2_, 5 μCi ^32^P-γATP, 10 μM ATP, 1 μg casein) and incubated at 30°C for 25 minutes. To remove HA-Nak1 following kinase reactions, the reactions were spun at 1000 rpm in a microfuge for 1 min, and the sepharose beads were removed. Samples boiled in Laemmli sample buffer for 5 min and resolved by SDS-PAGE; gels were stained with Commassie blue and visualized by autoradiography.
